# Persistence of antibodies 20 y after vaccination with a combined hepatitis A and B vaccine

**DOI:** 10.1080/21645515.2016.1274473

**Published:** 2017-03-10

**Authors:** Pierre Van Damme, Geert Leroux-Roels, P. Suryakiran, Nicolas Folschweiller, Olivier Van Der Meeren

**Affiliations:** a Centre for the Evaluation of Vaccination, Vaccine and Infectious Disease Institute, University of Antwerp, Antwerp, Belgium; b Center for Vaccinology, Ghent University Hospital, Ghent, Belgium; c GSK Pharmaceuticals, Mumbai, India; d GSK Vaccines, Wavre, Belgium

**Keywords:** Hepatitis A, Hepatitis B, Immune memory, Long-term, Persistence, Vaccination

## Abstract

Vaccination is the most effective and well-tolerated method of conferring long-term protection against hepatitis A and B viruses (HAV; HBV). Long-term studies are required to characterize the duration of protection and need for boosters. Following primary immunization of 150 and 157 healthy adults with 3-doses of combined hepatitis A/hepatitis B vaccine (HAB; *Twinrix*™, GSK Vaccines, Belgium) at 0-1-6 months in 2 separate studies, we measured vaccine-induced antibody persistence against HAV and HBV annually for 20 y (Study A: NCT01000324; Study B: NCT01037114). Subjects with circulating anti-HAV antibodies < 15 mIU/mL or with anti-hepatitis B surface antigen < 10 mIU/mL were offered an additional monovalent hepatitis A and/or B vaccine dose (*Havrix*™/*Engerix*™-B, GSK Vaccines, Belgium). Applying the immunogenicity results from these studies, mathematical modeling predicted long-term persistence. After 20 y, 18 and 25 subjects in studies A and B, respectively, comprised the long-term according-to-protocol cohort for immunogenicity; 100% and 96.0% retained anti-HAV antibodies ≥ 15 mIU/mL, respectively; 94.4% and 92.0% had anti-HBs antibodies ≥ 10 mIU/mL, respectively. Between Years 16–20, 4 subjects who received a challenge dose of monovalent hepatitis A vaccine (N = 2) or hepatitis B vaccine (N = 2), all mounted a strong anamnestic response suggestive of immune memory despite low antibody levels. Mathematical modeling predicts that 40 y after vaccination ≥ 97% vaccinees will maintain anti-HAV ≥ 15 mIU/mL and ≥ 50% vaccinees will retain anti-HBs ≥ 10 mIU/mL. Immunogenicity data confirm that primary immunization with 3-doses of HAB induces persisting anti-HAV and anti-HBs specific antibodies in most adults for up to 20 y; mathematical modeling predicts even longer-term protection.

## Introduction

Infections caused by the hepatitis A virus (HAV) and hepatitis B virus (HBV), which occur across the globe, are associated with significant morbidity and mortality, as well as inflicting a considerable healthcare burden.^1-3^ Vaccination is the most effective method of conferring long-term protection against both of these viruses and, together with the improved sanitation and hygiene has resulted in a steady reduction in global infection.^4-6^

Monovalent vaccines against hepatitis A and B are immunogenic and well tolerated^7-9^ with long-term immunogenic benefits observed in clinical studies with up to 20 y follow-up.^10-13^ Although hepatitis B post-vaccination titers ≥ 10mIU/mL are well established as guaranteeing long-term immunity and protection, the anamnestic responses seen in individuals with lower or non-detectable antibody titers ensure that protection is maintained.^14,15^ For hepatitis A, as the inactivated vaccines induce early post-vaccination circulating antibodies 100- to 1000-fold higher than those associated with clinical protection,^14^ and as anti-HAV titers following vaccination are around 5–6000 mIU/mL, but fall rapidly to 500–1000, a subject is considered protected after vaccination when antibodies are detectable. Nevertheless, a loss of detectable antibodies with time does not necessarily mean loss of protection. Due to the considerable overlap of risk factors and areas of high endemicity for both diseases, a combined vaccine against both viruses represents a pragmatic approach that reduces the number of vaccine administrations, in particular for travelers.^16^

Clinical development of the combined hepatitis A and B vaccine (HAB; *Twinrix*™ Adult; GSK Vaccines, Belgium), containing 720 EL.U inactivated HAV antigen and 20 µg hepatitis B surface antigen (HBs), was initiated in the 1990s.^7,9^ Nowadays, this bivalent vaccine is widely available, with a safety and immunogenicity profile demonstrated as comparable to the respective monovalent vaccines alone.^17^

In 1992–1993, 2 open phase IV clinical trials comparing the safety and immunogenicity of primary vaccination of healthy adults with 3 doses of the HAB vaccine were initiated.^16,18,19^ One month after completing the primary vaccination course (at Month 7), all subjects in both studies were seropositive for anti-HAV antibodies and had anti-HBs antibodies ≥ 10mIU/mL).^16,18,19^ These early trials were extended to allow for the annual collection of blood samples, and have subsequently demonstrated long-term antibody persistence after 6, 10 and 15 y.^20-22^ However, as the exact duration of this protection and the need for booster dosing are not known, even longer follow-up is required. In addition to the clinical observations, as adopted for the hepatitis A monovalent vaccine,^23^ such data can be used for mathematical modeling to predict long-term antibody persistence.

This manuscript now describes immunogenicity results obtained up to 20 y after primary vaccination with the HAB vaccine using the phase IV cohorts described earlier including the assessment of immune memory in subjects whose antibody levels had dropped below pre-defined cut-offs. In addition, these immunogenicity data were applied to mathematical models in an attempt to predict the long-term persistence of the immunity conferred by the combined HAB vaccine.

## Results

The primary vaccination studies were undertaken between October 1993 and June 1994 (Study A) and November 1993 and August 1994 (Study B), with the Year 16–20 follow-up visits taking place between November 2009 and July 2015 (Study A) and January 2010 and March 2014 (Study B).

### Study population

**Figure 1.**
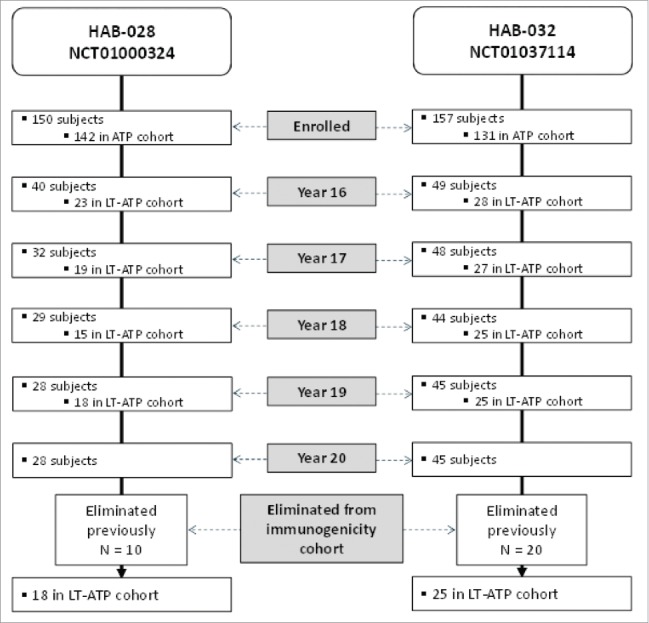
Subject disposition. LT-ATP: Long-term according-to-protocol (The LT-ATP cohort for immunogenicity included all subjects who were included in the ATP immunogenicity analysis in the primary study, for whom serology results were available for that blood sampling visit and who had not been eliminated due to any protocol violations).

Twenty years after primary vaccination, 28 subjects in Study A and 45 subjects in Study B returned for blood sampling (Fig. 1). The long-term according-to-protocol (LT-ATP) cohort for immunogenicity was restricted to fully vaccinated subjects who were seronegative before vaccination. In addition, data were censored from subjects who received an additional dose of monovalent hepatitis A or B vaccine from the time of re-vaccination, or who had deviations in their planned blood sample schedule. Therefore, at Year 20, 18 subjects in Study A and 25 subjects in Study B comprised the LT-ATP cohort at year 20.

The mean ages of the returning subjects were 39.4 y (range 38–41) in Study A and 42.3 y (range 38–62) in Study B. The majority of subjects in both studies were female (Study A: 78%; Study B: 80%) and Caucasian (Study A: 94%; Study B: 100%). These demographics were comparable with the LT-total (Study A mean age: 39.6 y; 82% female; 93% Caucasian; Study B mean age: 41.7 y; 80% female; 100% Caucasian) and with those in the original primary vaccination studies were comparable with respect to sex (82% in Study A and 81% in Study B were female) and race.^16, 18,19^

As shown in Table S1, the immune responses at Month 7 post-primary dosing were of the same magnitude in the Year 20 ATP cohort (anti-HAV and anti-HBs antibodies geometric mean concentrations (GMCs) as in the original primary vaccination cohorts at Month 7 for both studies (overlapping confidence intervals [CI]).

### Immunogenicity

#### Long-term antibody persistence 20 y after primary vaccination

Table 1 shows the percentage of subjects in the LT-ATP population who were seropositive (cut-off ≥ 33 mIU/mL for the original assay or ≥ 15 mIU/mL for the assay used for the last 15 y) for anti-HAV antibodies at each yearly follow-up time period after primary vaccination with the combined HAB vaccine in Studies A and B. Between Years 16 and 20 all remaining subjects in Study A and ≥ 96.0% subjects remaining in Study B were seropositive for anti-HAV antibodies. Over the same time period, all LT-total cohort subjects in Study A and ≥ 95.5% in Study B were seropositive for anti-HAV antibodies (Table S2). It should be noted that this represents a biased evaluation of long-term seroprotection as subjects who were re-vaccinated due to low titers were eliminated from the ATP cohort analysis, but not from the LT-total cohort.

**Table 1.** Percentage of subjects seropositive for anti-HAV antibodies at each yearly follow-up point during the 20 y follow-up period (LT-ATP cohort for immunogenicity).

	Study A			Study B		
Time point	N	Seropositive	95% CI	N	Seropositive	95% CI
**Cut-off ≥ 33 mIU/mL (*Enzymum*™ ELISA kit, Boehringer Mannheim)**						
**PIII(M7)**	107	100.0	96.6;100.0	116	100.0	96.9;100.0
**PIII(M18)**	86	100.0	95.8;100.0	93	100.0	96.1;100.0
**Year 2**	75	100.0	95.2;100.0	86	100.0	95.8;100.0
**Year 3**	54	100.0	93.4;100.0	80	100.0	95.5;100.0
**Year 4**	49	100.0	92.7;100.0	71	100.0	94.9;100.0
**Year 5**	43	100.0	91.8;100.0	65	100.0	94.5;100.0
**Year 6**	40	100.0	91.2;100.0	47	100.0	92.5;100.0
**Cut-off ≥ 15 mIU/mL (*Enzygnost*™ EIA kit, DADE Behring)**						
Year 6^*^	40	100.0	91.2;100.0	51	100.0	93.0;100.0
**Year 7**	35	100.0	90.0;100.0	45	100.0	92.1;100.0
**Year 8**	35	100.0	90.0;100.0	42	100.0	91.6;100.0
**Year 9**	28	100.0	87.7;100.0	40	100.0	91.2;100.0
**Year 10**	29	100.0	88.1;100.0	34	100.0	89.7;100.0
**Year 11**	25	100.0	86.3;100.0	33	100.0	89.4;100.0
**Year 12**	28	100.0	87.7;100.0	33	100.0	89.4;100.0
**Year 13**	23	100.0	85.2;100.0	32	100.0	89.1;100.0
**Year 14**	24	100.0	85.8;100.0	30	100.0	88.4;100.0
**Year 15**	31	100.0	88.8;100.0	29	100.0	88.1;100.0
**Year 16**	23	100.0	85.2;100.0	28	100.0	87.7;100.0
**Year 17**	19	100.0	82.4;100.0	27	96.3	81.0;99.9
**Year 18**	10	100.0	69.2;100.0	25	100.0	86.3;100.0
**Year 19**	17	100.0	80.5;100.0	25	96.0	79.6;99.9
**Year 20**	18	100.0	81.5;100.0	25	96.0	79.6;99.9

N: number of subjects with available results; CI: confidence interval; ELISA: enzyme-linked immunosorbent assay; EIA: enzyme-linked immunoassay; LT-ATP: Long-term according-to-protocol; HAV: hepatitis A virus

* The anti-HAV assay was changed at Year 7, and the previous timepoint was re-tested for bridging purposes

**Figure 2.**
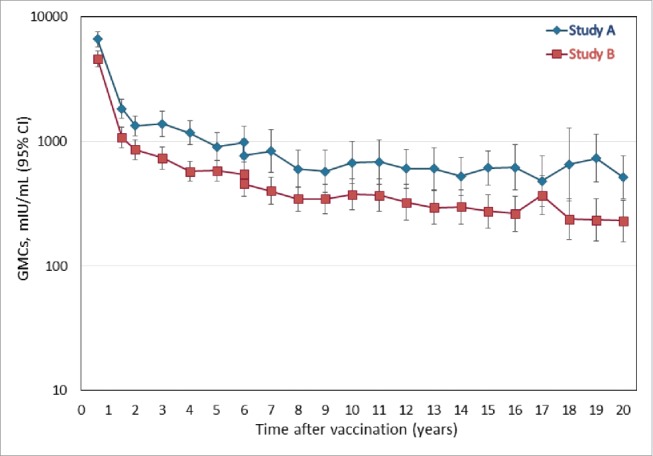
Anti-HAV antibody geometric mean concentration at each yearly follow-up point during the 20 y follow-up period (LT-ATP cohort for immunogenicity).

Figure 2 presents the evolution of the anti-HAV antibody GMCs at the yearly timepoint in the LT-ATP cohort. The leveling off seen at the Year 15 timepoint [16] appears to be maintained 20 y after primary vaccination. At the Year 20 timepoint, the anti-HAV antibody GMCs in the LT-ATP cohort were 511.9 mIU/mL (95% CI: 343.8; 762.0) in Study A and 229.3 mIU/mL (95% CI 157.1; 334.6) in Study B. The corresponding values in the LT-total cohort were 487.9 mIU/mL (95% CI 339.6; 701.0) and 237.4 mIU/mL (95% CI 168.4; 334.9), respectively (Table S3).

Table 2 shows the percentage of subjects in the LT-ATP population who had anti-HBs titers ≥ 10 mIU/mL or seropositive (cut-offs for each assay shown in the Table) for anti-HBs antibodies each year for 20 y after HAB primary vaccination. Between Years 16 and 20, ≥ 87.0% subjects in Study A and ≥ 92.0% in Study B were seropositive for anti-HBs antibodies. Over the same time period, the corresponding percentages in the LT-total cohort subjects were ≥ 96.4% and ≥ 93.0%, respectively (Table S4). As for HAV, subjects who were revaccinated due to low titers were eliminated from ATP but not from LT total cohort.

**Table 2.** Percentage of subjects seroprotected (≥ 10 mIU/mL) and seropositive for anti-HBs antibodies at each yearly follow-up point during the 20 y follow-up period (LT-ATP cohort for immunogenicity).

	Study A					Study B				
		Seropositive		≥ 10 mIU/mL			Seropositive		≥ 10 mIU/mL	
Time point	N	%	95% CI	%	95% CI	N	%	95% CI	%	95% CI
**Cut-off ≥ 1.0 mIU/mL (RIA kits; Abbott)**										
**PIII(M7)**	107	100.0	96.6; 100.0	100.0	96.6; 100.0	116	100.0	96.9; 100.0	100.0	96.9; 100.0
**PIII(M18)**	86	100.0	95.8; 100.0	98.8	93.7; 100.0	92	97.8	92.4; 99.7	95.7	89.2; 98.8
**Year 2**	75	100.0	95.2; 100.0	97.3	90.7; 99.7	86	100.0	95.8; 100.0	97.7	91.9; 99.7
**Year 3**	54	98.1	90.1; 100.0	96.3	87.3; 99.5	80	100.0	95.5; 100.0	98.8	93.2; 100.0
**Year 4**	49	98.0	89.1; 99.9	93.9	83.1; 98.7	71	100.0	94.9; 100.0	95.8	88.1; 99.1
**Year 5**	43	97.7	87.7; 99.9	93.0	80.9; 98.5	65	100.0	94.5; 100.0	96.9	89.3; 99.6
**Year 6**	40	95.0	83.1; 99.4	95.0	83.1; 99.4	47	100.0	92.5; 100.0	89.4	76.9; 96.5
**Cut-off ≥ 3.3 mIU/mL (EIA kits; Abbott)**										
Year 6^*^	40	95.0	83.1; 99.4	95.0	83.1; 99.4	51	98.0	89.6; 100.0	96.1	86.5; 99.5
**Year 7**	35	91.4	76.9; 98.2	91.4	76.9; 98.2	45	100.0	92.1; 100.0	97.8	88.2; 99.9
**Year 8**	35	91.4	76.9; 98.2	91.4	76.9; 98.2	42	100.0	91.6; 100.0	100	91.6; 100.0
**Year 9**	28	89.3	71.8; 97.7	89.3	71.8; 97.7	40	100.0	91.2; 100.0	97.5	86.8; 99.9
**Year 10**	29	89.7	72.6; 97.8	86.2	68.3; 96.1	34	100.0	89.7; 100.0	94.1	80.3; 99.3
**Year 11**	25	92.0	74.0; 99.0	92.0	74.0; 99.0	33	100.0	89.4; 100.0	100	89.4; 100.0
**Year 12**	28	89.3	71.8; 97.7	89.3	71.8; 97.7	33	100.0	89.4; 100.0	97.0	84.2; 99.9
**Cut-off ≥ 3.3 mIU/mL (ELISA kits; in-house)**										
**Year 13**	23	87.0	66.4; 97.2	87.0	66.4; 97.2	32	100.0	89.1; 100.0	100.0	89.1; 100.0
**Year 14**	24	87.5	67.6; 97.3	87.5	67.6; 97.3	30	96.7	82.8; 99.9	96.7	82.8; 99.9
**Cut-off ≥ 6.2 mIU/mL (CLIA kits)**										
Year 14*	24	87.5	67.6; 97.3	87.5	67.6; 97.3	28	96.4	81.7; 99.9	96.4	81.7; 99.9
**Year 15**	31	90.3	74.2; 98.0	90.3	74.2; 98.0	29	96.6	82.2; 99.9	96.6	82.2; 99.9
**Year 16**	23	87.0	66.4; 97.2	87.0	66.4; 97.2	28	96.4	81.7; 99.9	92.9	76.5; 99.1
**Year 17**	19	89.5	66.9; 98.7	89.5	66.9; 98.7	27	92.6	75.7; 99.1	92.6	75.7; 99.1
**Year 18**	10	90.0	55.5; 99.7	90.0	55.5; 99.7	25	92.0	74.0; 99.0	92.0	74.0; 99.0
**Year 19**	18	94.4	72.7; 99.9	94.4	72.7; 99.9	25	92.0	74.0; 99.0	92.0	74.0; 99.0
**Year 20**	18	94.4	72.7; 99.9	94.4	72.7; 99.9	25	92.0	74.0; 99.0	92.0	74.0; 99.0

N: number of subjects with available results; CI: confidence interval; RIA: radioimmunoassay; EIA: enzyme-linked immunoassay; ELISA: enzyme-linked immunosorbent assay; CLIA: chemiluminescence assay; LT-ATP: Long-term according-to-protocol

* The anti-HBs assay was changed at Years 7 and 15, and the previous timepoints were re-tested for bridging purposes

**Figure 3.**
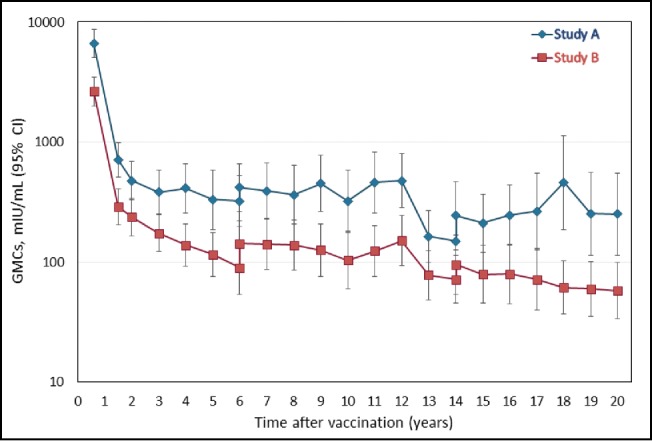
Anti-HBs antibody geometric mean concentration at each yearly follow-up point during the 20 y follow-up period (LT-ATP cohort for immunogenicity).

As shown in Fig. 3, the leveling off in anti-HBs antibody GMCs observed at the Year 15 timepoint,^22^ was maintained 20 y after primary vaccination. At the Year 20 timepoint, the anti-HBs antibody GMCs in the LT-ATP cohort were 249.9 mIU/mL (95% CI 114.2; 546.5) in Study A and 57.7 mIU/mL (95% CI 33.6; 99.1) in Study B. The corresponding values in the LT-total cohort were 304.9 mIU/mL (95% CI 176.8; 525.8) and 83.5 mIU/mL (95% CI 48.9; 142.7), respectively (Table S5).

#### Anamnestic response to a challenge dose of hepatitis A or/and hepatitis B vaccine in subjects with anti-HAV antibody concentration <15 mIU/mL or/and anti-HBs antibody concentration ≤10 mIU/mL

Details of the subjects who required additional challenge doses are presented in Table 3. During the Year 16 to 20 time period reported here, 2 subjects in Study B had anti-HAV concentrations <15 mIU/mL (at Years 17 and 19), and received an additional dose of hepatitis A vaccine at Years 18 and 20, respectively. Within this time frame, 2 subjects lost circulating anti-HBs antibodies (at Year 18 for the Study A subject and at Year 15 for the Study B subject) and received a challenge dose of hepatitis B vaccine at Year 19 and Year 16, respectively. All subjects mounted strong anamnestic responses following the booster doses of monovalent hepatitis A or hepatitis B vaccines (Table 3). Indeed, as compared with pre-challenge, the 30 day post-challenge GMCs were 55–135 fold higher after the hepatitis A vaccine and 1035–3930 fold higher following the hepatitis B vaccine.

**Table 3.** Individual immune responses to a challenge dose of hepatitis A or/and hepatitis B vaccine during years 16 to 20.

	Antibody concentration (mIU/mL)				
Study	At timepoint determining eligibility (Year)	Pre-challenge (Year)	Day 14 post-challenge	Day 30 post-challenge	Anamnestic response
***Response to Hep A vaccine challenge dose***					
**B**	<15 (Year 17)	25 (Year 19)	3421	3389	Yes^*^
**B**	<15 (Year 19)	19 (Year 20)	1096	1060	Yes^*^
***Response to Hep B vaccine challenge dose***					
**A**	13.26 (Year 18)^#^	12.27 (Year 19)	21926.00	12736.00	Yes^**^
**B**	10.97 (Year 15)^#^	7.86 (Year 16)	50640.00	31442.00	Yes^**^

# The eligibility checks for these subjects were originally performed using ELISA, however the concentrations presented in the table are the CLIA re-test results

* Anti-HAV anamnestic response defined as anti-HAV antibody concentrations one month post-challenge: either ≥ 15 mIU/mL in subjects, seronegative at pre-challenge; or ≥ 2-fold increase in subjects with pre-challenge anti-HAV antibody concentrations ≥ 100 mIU/mL; or ≥ 4-fold increase in seropositive subjects having pre-challenge anti-HAV antibody concentrations <100 mIU/mL

** Anti-HBs anamnestic response defined as anti-HBs antibody concentrations one month post-challenge: either ≥ 10 mIU/mL in subjects seronegative at pre-challenge; or ≥ 4-fold increase in subjects seropositive at pre-challenge

### Modeling results

**Figure 4.**
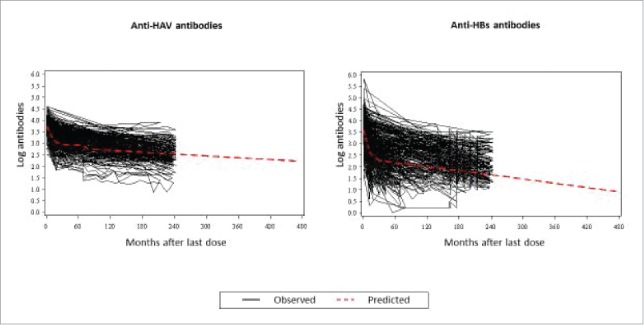
Observed individual profiles and population-averaged estimation of anti-HAV and anti-HBs levels.

Observed data at Year 20 from 70 subjects (for anti-HAV predictions) and 57 (for anti-HBs predictions) were used in the model to predict long-term antibody persistence (Fig. 4). Table 4 shows the distribution (percentile) of predicted anti-HAV and anti-HBs levels up to Year 40. Using ≥ 15mIU/mL as the seropositivity cut-off for anti-HAV antibodies, the model predicts that ≥ 97% subjects will remain seropositive at Year 40 (95% CI: 94.28; 98.93) (Table 4). For anti-HBs antibodies, at Year 40 ≥ 50% subjects are predicted to remain ≥ 10 mIU/mL (95% CI: 32.46; 70.52) (Table 4).

**Table 4.** Distribution (percentiles) of predicted Anti-HAV/ Anti-HBs levels and predicted proportion of subjects above pre-specified cut-offs.

		Distribution of predicted levels										
	Study month (Year)	N	3%	5%	10%	25%	50%	75%	90%	95%	97%	Predicted proportion (95% confidence intervals)
**Anti-HAV**												**≥ 15 mIU/mL**
	228 (Y19)	68	41.54	58.66	105.32	176.58	372.50	786.13	1694.55	1942.37	2071.76	
	240 (Y20)	70	41.27	53.58	124.95	169.19	316.94	727.49	1607.43	1907.15	2081.89	98.57 (95.38;100.0)
	300 (Y25)	280	39.98	49.97	82.58	154.78	291.20	582.39	979.19	1499.98	1837.83	98.93 (97.50;100.0)
	360 (Y30)	280	29.64	38.58	62.12	126.26	238.36	486.53	916.82	1497.86	1915.15	98.93 (97.14;100.0)
	420 (Y35)	280	21.00	31.59	50.04	100.26	195.42	413.68	826.15	1388.89	1898.64	97.50 (95.36;99.29)
	480 (Y40)	280	15.63	23.30	39.14	80.31	161.64	349.26	699.62	1283.91	2029.46	97.14 (94.28;98.93)
**Anti-HBs**												**≥ 10 mIU/mL**
	228 (Y19)	61	12.17	15.99	23.28	40.57	97.21	292.09	822.46	1030.53	1482.75	
	240 (Y20)	59	11.20	14.09	21.43	36.55	91.06	256.88	784.94	1061.87	1275.77	100.00 (98.15;100.00)
	300 (Y25)	268	0.63	0.81	2.31	10.01	35.16	98.47	319.30	618.25	964.39	75.00 (62.69;86.19)
	360 (Y30)	268	0.28	0.49	1.41	6.45	22.95	65.65	234.99	434.29	615.95	67.16 (51.86;81.34)
	420 (Y35)	268	0.15	0.29	0.86	4.42	15.53	44.20	157.91	367.09	496.21	59.33 (44.03;76.49)
	480 (Y40)	268	0.10	0.17	0.52	2.75	10.04	30.18	111.36	262.60	429.54	50.37 (32.46;70.52)

### Safety and reactogenicity

No adverse events were recorded after the 2 challenge doses of hepatitis A vaccine. The subject in Study A who received the challenge dose of hepatitis B vaccine at Year 19 reported 2 unsolicited symptoms (musculoskeletal stiffness and oropharyngeal pain) during the 31-day follow-up period; the musculoskeletal stiffness was considered causally related to vaccination. The subject in Study B who received a hepatitis B challenge dose at Year 16 recorded an unsolicited symptom (back pain) which was not considered to be related to vaccination. No serious adverse events were reported during the Year 16 to 20 time period in either of the 2 long-term studies.

## Discussion

Many studies have already demonstrated long-term immune persistence after monovalent and combined hepatitis A and B vaccination in children, adolescents and adults.^10–13,22^ However, even longer term studies are needed to characterize antibody persistence and booster requirements for residents of or travelers to endemic regions. The studies described here have undertaken the longest follow-up to date of the combined HAB vaccine, for 20 y after a 3 dose primary vaccination course in healthy adults.

These extended studies showed that the HAB vaccine induced prolonged high levels of immunogenicity, with at least 96% of subjects remaining seropositive for anti-HAV antibodies and at least 92% remaining ≥ 10 mIU/mL for anti-HBs antibodies 20 y after vaccination.

As has been known for many years,^24^ the pattern of anti-HAV antibody evolution following primary vaccination consists of an initial steep decline followed by a second slower reduction. We observed a steep fall in both anti-HAV and anti-HBs antibodies up to Month 12 in both studies, followed by an apparent stabilization until Year 15.^22^ Our current results confirm that the vast majority of subjects still present circulating antibodies after 20 y, and suggest that the shallow decrease in GMC is maintained [anti-HAV antibody GMCs: 229.3–511.9 mIU/mL; anti-HBs GMCs: 57.7–249.9 mIU/mL]. Indeed both curves were very similar to the one observed after 20 y of follow-up after primary vaccination of adults with monovalent hepatitis A vaccine.^10^ One point of note was that the anti-HAV GMCs were higher in Study A than Study B (significant differences [non-overlapping CIs] from Year 16 and Years 18–20) and were significantly higher from Years 16–20 for anti-HBs GMCs. Such a trend was also commented upon at Year 15, when the differences were attributed to likely inconsistencies in the study cohorts and natural variations in GMC.^22^

The first observations from these clinical trials had suggested that protection would last 5–10 y.^16,18,19^ However, by applying these data for hepatitis A vaccine in mathematical models, it was estimated that anti-HAV antibody titers after a complete primary course would persist for 25 y in 95% of vacinees.^23^ Our current data, using the HAB vaccine, collected up to 20 y after vaccination, confirm that most subjects still present circulating antibodies at Year 20. However, by applying these immunogenicity data for mathematical modeling predicts that at Year 40, ≥ 97% subjects will remain seropositive for anti-HAV antibodies and ≥ 50% subjects will remain ≥ 10 mIU/mL for anti-HBs antibodies. These results are consistent with other studies of long-term anti-HAV and anti-HBs persistence in adults following monovalent vaccine administration.^10,13,25^ The percentage of subjects predicted to remain ≥ 10 mIU/mL for anti-HBs does appear to be low. Indeed, although many studies with monovalent hepatitis B vaccine administered to various populations,^26,27^ or combined hepatitis B, for example within the hexavalent diphtheria-tetanus-acellular pertussis-hepatitis B-inactivated polio/ *Haemophilus influenzae* (DTPa-HBV-IPV/Hib) vaccine administered to infants,^28,29^ show long-term persistence, a decrease of seroprotection rates with time is observed. Nevertheless, as we observed here, when a booster is administered, all subjects demonstrate anamnestic response suggesting the presence of immune memory despite the disparity of circulating antibodies.^26-28^

The robust anamnestic responses to both vaccines observed at the Year 15 timepoint,^22^ suggested that immune memory persists in the long-term. This was again demonstrated in the Year 16–20 follow up studies, when the 4 subjects who required either a challenge dose of hepatitis A vaccine (n = 2) at Years 19 or 20, or hepatitis B vaccine (n = 2) at Years 16 and 19 all mounted strong anamnestic responses.

This trial was limited because all subjects received the bivalent vaccine in the initial primary vaccination study; there were no monovalent vaccine controls.^16,18,19^ Nevertheless, our results are consistent with separate studies where long-term persistence after monovalent primary vaccination has been observed.^10-13^ Another limitation of these long-term studies is the inevitable subject attrition. At Year 20, 28/150 subjects (18.6%) in Study A and 45/156 subjects (28.8%) were included in the total cohort (18/142 [12.7%] and 25/131 [19%], respectively, in the LT-ATP). Such attrition could bias the demographic characteristics of the subjects in the follow-up studies. For example, if fewer younger subjects with better immune responses dropped out from the studies, then the observed protection levels would be greater than a population which included a higher proportion of older subjects. However, the demographics of the subjects returning at Year 20 were comparable with those in the original primary vaccination studies, which also each had over 80% of female subjects. Specifically, the mean ages of the subjects returning at Year 20 (39.6 and 41.7 y) were exactly 20 y greater than those at baseline in the original studies (19.6 and 21.7 y, respectively [16]), suggesting that returning population included a similar distribution of ages. Finally, considering the wide confidence intervals we cannot exclude an overestimation of the percentage of subjects seroconverted/seroprotected.

In conclusion, these studies suggest that a complete 3-dose regimen of the combined HAB vaccine induces long-term protection against HAV and HBV in the vast majority of adults up to 20 y after vaccination, which is at least as good as that of the respective monovalent vaccines. Further, mathematical modeling predicts that 40 y after vaccination, ≥ 97% and ≥ 50% vaccinees will remain seropositive for anti-HAV and ≥ 10 mIU/mL for anti-HBs antibodies, respectively. In the small number of subjects who lose circulating antibodies, strong anamnestic responses to challenge doses, indicate that a 3-dose primary vaccine schedule confers long-term immune memory.

## Materials and methods

### Study design and subjects

Two phase-IV, open-label, single group, single center follow-up studies (Study A: NCT01000324; Study B: NCT01037114) were undertaken in Belgium to evaluate the long-term persistence of antibodies against hepatitis A and B. In the original primary vaccination studies, healthy adults aged 17–39 y (Study A),^16,18^ or 17–43 y (Study B)^16,19^ received 3 doses of the combined HAB vaccine at 0, 1 and 6 months. All subjects were required to be negative for anti-HAV, anti-HBc and/or HBs at screening.

In the follow-up studies described in this manuscript, eligible subjects were invited for yearly follow-up appointments for blood sampling from Year 16 until Year 20. Both studies were approved by their respective Ethics Review Committees and were conducted in accordance with the Declaration of Helsinki and Good Clinical Practice guidelines. Returning subjects provided written informed consent at each follow-up time-point before any interventions were undertaken.

### Vaccines

In the original primary vaccination studies 3 doses of the combined HAB vaccine [*Twinrix*™ *Adult* (GSK Vaccines, Belgium 720 El.U HAV and 20µg HBsAg)] were administered to all subjects.^16,18,19^ In the follow-up studies described here, subjects who became seronegative (anti-HAV antibody concentration <15mIU/mL and/or anti-HBs antibody concentrations <10 mIU/mL) between Years 16 and 20, were offered a booster vaccine dose of monovalent hepatitis A vaccine (*Havrix*™; ≥ 1440 El.U/mL hepatitis A antigen) or/and monovalent hepatitis B vaccine (*Engerix-B*™; 20µg HBsAg) at their next visit. Subjects receiving a booster dose were subsequently excluded from the ATP cohort.

The booster dose challenge was administered as a deep intramuscular injection (needle length: 25 mm) in the non-dominant deltoid muscle. However, if both vaccines were required, the hepatitis B vaccine was administered in the dominant arm.

### Assessments

Blood samples were collected from each subject at the annual follow-up visits for immunogenicity analysis. Over the course of the 20 y follow-up, several assay changes were required and details of the assays used until Year 15 are described by Van Damme et al (2012).^22^ From Years 16 to 20, anti-HAV antibodies were assessed using a commercially available enzyme-linked immunoassay (*Enzygnost™*, Siemens Healthcare, Germany; seropositivity cut-off: ≥ 15 mIU/mL). Anti-HBs antibody concentrations were determined using a Chemiluminescence immunoassay (*Centaur*™, Siemens Healthcare, Germany) with a 6.2 mIU/mL seropositivity cut-off; concentrations ≥ 10 mIU/mL were considered to be seroprotective. The Geometric Mean Titres (GMTs) calculations were performed by taking the anti-log of the mean of the log titer transformations. Antibody titres below the cut-off of the assay were given an arbitrary value of half the cut-off for the purpose of GMT calculation.

For subjects who required a booster challenge dose, based on the blood samples they provided at their previous annual visit, blood samples were taken immediately before and 14 and 30 d after vaccine challenge. The anti-HAV and anti-HBs antibody concentrations were determined as described above. The anti-HAV anamnestic response was defined as having anti-HAV antibody concentrations one month post-challenge: either ≥ 15 mIU/mL in subjects, seronegative at pre-challenge; or ≥ 2-fold increase in subjects with pre-challenge anti-HAV antibody concentrations ≥ 100 mIU/mL; or ≥ 4-fold increase in seropositive subjects having pre-challenge anti-HAV antibody concentrations <100 mIU/mL. The anti-HBs anamnestic response was defined as having anti-HBs antibody concentrations one month post-challenge: either ≥ 10 mIU/mL in subjects seronegative at pre-challenge; or ≥ 4-fold increase in subjects seropositive at the pre-challenge timepoint.

Solicited local and general adverse events after the vaccine challenge dose were recorded for 4 d and unsolicited adverse events for 30 d post-challenge. Serious adverse events, which were considered to be causally related to vaccination were recorded throughout the entire study period.

### Statistical analyses

The immunogenicity analyses were performed on the LT-ATP and LT-total cohorts. All subjects included in the total cohort in the primary study and who attended annual follow-up visits at Years 16–20 constituted the LT-total cohort for that year. The LT-ATP cohort included all subjects from the ATP cohort in the primary study who had not been excluded from the ATP cohort at any subsequent follow-ups, had complied with the specific year blood sampling intervals and had not received any additional hepatitis A or B vaccines or recorded any abnormal increases in anti-HAV or anti-HBs antibody concentrations since the previous time point. Subjects who required a booster dose remained included in the ATP cohort. After receiving the booster dose, their data were censored from the ATP analyses. Additional post-hoc analyses were undertaken to calculate the Month 7 post-primary immune responses in the subjects included in the Year 20 ATP and total cohorts.

The statistical analyses were performed using the Statistical Analysis Systems (SAS) version 9.2 (SAS Institute Inc., Cary, NC).

### Mathematical modeling for long-term predictions

The population included in the mathematical modeling was slightly different to those providing the previously described immunogenicity results: some subjects who were excluded from the immunogenicity analysis for reasons not impacting upon antibody persistence were included in the model. In addition, the mathematical modeling was not previously planned in the original protocol.

A linear-mixed model, which included an indicator variable, was used to predict seropositivity rates (anti-HAV cut-off: ≥ 15 mIU/mL; anti-HBs cut-off ≥ 6.2 mIU/mL) at 25, 30, 35 and 40 y after vaccination. The indicator variable was included to distinguish between antibody levels measured with different assays (RIA, ELISA, CLIA for HBs) and (GSK-in-house, New assay for HAV), as different assays/cut offs were used at different time points in the primary studies. The estimation period for Anti-HBs for both studies (HAB-028 and HAB-032) consisted of measurements based on: the RIA assay for the first 72 months; the ELISA assay from Month 72 until Month 216; the CLIA assay Month 168 until Month 240. The estimation period for Anti-HAV for both studies consisted of measurements based on the GSK-in-house assay for the first 72 months and the new assay from Month 72 until Month 240. The model was fitted using all available data through Year 20. The Akaike Information Criterion (AIC) was used for model selection and goodness of fit was assessed using standard diagnostic tools for linear mixed models.

The linear model used 2 change-points at 6 and 24 months after the last vaccine dose. These change points were selected, based on those used in the modeling study for long-term HAV prediction published by Theeten et al 2015.^10^

As described previously, data were processed using SAS.^10,30^ Representation of the mean trend in observed antibody levels (Fig. 4) and calculation of the coefficient of simple determination (R2) and D index of agreement (data not shown) followed the methods described by Hens et al 2014.^30^ The proportion of subjects with a predicted titer value above the pre-specified cut-off were estimated. The uncertainty of the predicted proportions was assessed using a nonparametric bootstrap method to obtain the 95% CI on the predictions. Data was re-sampled (with re-sampling by individual) and the model was refitted to the re-sampled data yielding a bootstrap-estimate for the proportion immune response for anti-HBs and anti-HAV. This process was repeated 1000 times and the 2.5% and 97.5% percentiles of the resulting estimates were used as lower and upper CI, respectively.

## Registration

ClinicalTrials.gov number: NCT01000324 and NCT01037114.

## Trademark statement

*Engerix-B*™, *Havrix*™ and *Twinrix*™ are trademarks of the GlaxoSmithKline group of companies.

*Enzygnost™* and *Centaur™* are trademarks of Siemens Healthcare.

*Enzymum* is a trademark of Boehringer Mannheim.

## Abbreviations

ATP: According-to-protocol
CI: confidence interval
CLIA: chemiluminescence assay
EIA: enzyme immunoassay
ELISA: enzyme-linked immunosorbent assay
EL.U: ELISA units
GMC: geometric mean concentration
HAB: combined hepatitis A and hepatitis B vaccine
HAV: hepatitis A virus
HBs: Hepatitis B surface antigen
HBV: hepatitis B virus
LT: long-term
RIA: radioimmunoassay
